# When Obesity Hits the Kidneys: Sex‐Specific Effects of Visceral Adiposity Indices on Diabetic Kidney Disease Risk

**DOI:** 10.1155/jnme/7057744

**Published:** 2026-07-20

**Authors:** Xuezhe Wang, Yi Kang, Qian Jin, Mengqi Zhou, Danwen Li, Xiaowen Li, Zhenjie Chen, Jingwei Zhou, Huijuan Zheng, Jie Lv, Yaoxian Wang

**Affiliations:** ^1^ Department of Nephrology and Endocrinology, Dongzhimen Hospital, Beijing University of Chinese Medicine, Beijing, China, bucm.edu.cn; ^2^ Graduate School, Beijing University of Chinese Medicine, Beijing, China, bucm.edu.cn; ^3^ Department of Integrative Oncology, China-Japan Friendship Hospital, Beijing, China, zryhyy.com.cn; ^4^ Department of Traditional Chinese Medicine, Beijing Puren Hospital, Beijing, China

**Keywords:** diabetes, diabetic kidney disease, sex-specific analysis, visceral obesity indices

## Abstract

**Background:**

Visceral obesity is a significant risk factor for metabolic disorders and is also an important risk factor for diabetic kidney disease (DKD). However, there is still insufficient research on the predictive value of different visceral obesity indices for DKD and the sex differences in this regard. This study aims to explore the correlations between the cardiac metabolic index (CMI), lipid accumulation product (LAP), triglyceride–glucose (TyG) index, visceral adiposity index (VAI), and the risks and mortality associated with DKD, with a focus on sex differences.

**Methods:**

This study utilized data from NHANES conducted from 2007 to 2016. Weighted logistic regression models were employed to investigate the relationships between four visceral obesity indices and DKD. Restricted cubic splines (RCSs) were also used to assess the dose–response relationship, and subgroup and interaction analyses were conducted. Receiver operating characteristic (ROC) curve analysis was conducted to evaluate the screening utility of each index for DKD risk identification. Kaplan–Meier (K–M) curves were used to analyze survival outcomes across different tertiles of the four visceral obesity indices.

**Results:**

A total of 6722 diabetic patients were included in the study, with 5301 in the diabetes‐only group and 1421 in the DKD group. All four visceral obesity indices were significantly positively correlated with the risk of DKD, exhibiting a nonlinear relationship, with notable sex differences. Compared to males, TyG and LAP showed a stronger correlation in females. The area under the curve (AUC) was generally higher in females. Furthermore, all four visceral obesity indices showed significantly lower all‐cause and cardiovascular mortality rates in the lowest tertile (Q1 group) compared to the higher tertiles (Q2‐Q3 groups).

**Conclusion:**

CMI, LAP, TyG, and VAI are significantly associated with the risk of DKD in diabetic patients, with notable differences between male and female populations. These metabolic indices may serve as important indicators for risk assessment and potential intervention strategies for DKD.

## 1. Introduction

Diabetic kidney disease (DKD) is one of the most common microvascular complications of diabetes, accounting for approximately 50% of the global chronic kidney disease cases [1]. It has become the leading cause of end‐stage renal disease (ESRD) worldwide [2]. DKD patients have a 20‐ to 40‐fold higher risk of cardiovascular disease (CVD) than diabetic patients without kidney disease, and most die from cardiovascular events before renal replacement therapy [3]. Traditional studies have mainly focused on the impact of glycemic control on DKD, but the cardiovascular–kidney–metabolic syndrome (CKM) proposed by the American Heart Association (AHA) in 2023 [4] further revealed that DKD is fundamentally a systemic disease driven by metabolic disturbances. Abnormal glucose and lipid metabolism damage the kidney by inducing oxidative stress, inflammation, and renin–angiotensin system activation, resulting in tubular fibrosis, glomerular hypertrophy, and sclerosis [5, 6]. Focusing on metabolic risk factors, obesity, as a core component of metabolic syndrome, has become a research hotspot due to its association with DKD.

Obesity is a significant risk factor for both diabetes and kidney disease [7, 8]. In particular, visceral obesity is an independent risk factor for Type 2 diabetes (T2DM), its complications, and even all‐cause mortality [9]. Traditional obesity indices have limitations: body mass index (BMI) cannot distinguish fat distribution and may misclassify muscular individuals, while waist circumference and waist‐to‐hip ratio reflect only body shape, not metabolic activity. Studies have also confirmed that the commonly used BMI is not a good predictor of chronic kidney disease [8, 10]. Although imaging‐based fat measurements are accurate and reliable, their high cost limits their widespread use in clinical practice [11, 12]. In recent years, several new indices for measuring visceral obesity have been developed, such as the cardiometabolic index (CMI) [13], lipid accumulation product (LAP) [13], triglyceride–glucose index (TyG) [14], and visceral adiposity index (VAI) [15]. These indices provide a potential for more accurate assessment of visceral obesity, yet there are still significant limitations: (1) Research has focused on individual indices, lacking comparisons across all four indices; (2) the predictive efficacy of different indices for DKD progression remains unclear; and (3) the impact of biological gender differences on these associations has not been fully elucidated. Moreover, studies on the association between these indices and DKD are still insufficient.

Based on this, the present study aims to systematically analyze the associations between four visceral obesity indices (CMI, LAP, TyG, and VAI) and DKD risk, conducting sex‐specific analyses to compare their predictive value and explore gender differences. The study seeks to provide new metabolic biomarkers and theoretical support for early warning and targeted interventions in DKD.

## 2. Methods

The National Health and Nutrition Examination Survey (NHANES) is a nationwide study conducted by the National Center for Health Statistics (NCHS), a division of the Centers for Disease Control and Prevention (CDC). Its purpose is to assess the health and nutritional status of the noninstitutionalized U.S. population (https://wwwn.cdc.gov/nchs/nhanes/). The study employs a complex, stratified multistage probability sampling design to select a nationally representative sample [16]. The NHANES study protocol has been formally approved by the CDC Institutional Review Board (IRB), and all participants voluntarily provide written informed consent. The study was conducted in accordance with the ethical principles of the Declaration of Helsinki. Due to the public nature of the NHANES database, research using its data does not require additional Institutional Review Board approval.

### 2.1. Study Design and Participants

**FIGURE 1:**
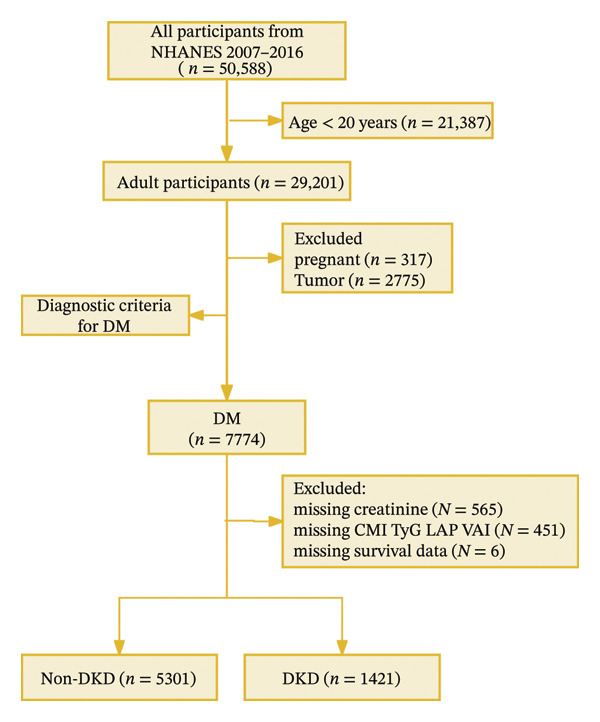
Study flowchart for the enrollment of study population. DM: diabetes mellitus; CMI: cardiometabolic index; LAP: lipid accumulation product; TyG: triglyceride–glucose index; VAI: visceral adiposity index; DKD: diabetic kidney disease.

The study is based on data from five consecutive NHANES cycles (2007‐2008, 2009‐2010, 2011‐2012, 2013‐2014, and 2015‐2016), initially including 50,488 participants. Individuals under 20 years of age (*N* = 21,387), pregnant women (*N* = 317), and those with a history of cancer (*N* = 2775) were excluded. Among the remaining participants, 7744 patients with diabetes were identified. After further excluding participants with missing data on serum creatinine (Scr), waist circumference, height, weight, high‐density lipoprotein cholesterol (HDL), triglycerides (TGs), and mortality status, a total of 6722 participants were included in the final analysis. Among these, 5301 had non‐diabetic kidney disease (non‐DKD) and 1421 had DKD (Figure 1).

### 2.2. Definitions of DKD and Visceral Obesity Indices

According to the standards of the American Diabetes Association and previous studies, the diagnosis of diabetes is based on (1) a diagnosis previously reported by a healthcare professional, or (2) a fasting blood glucose ≥ 7.0 mmol/L, or (3) a glycated hemoglobin (HbA1c) ≥ 6.5%, or (4) current treatment for diabetes (medications/insulin). The albumin‐to‐creatinine ratio (ACR) is calculated based on the urine albumin and creatinine ratio, and the glomerular filtration rate (eGFR) is calculated using the Chronic Kidney Disease Epidemiology Collaboration (CKD‐EPI) equation. An ACR ≥ 30 mg/g and/or an eGFR < 60 mL/min/1.73 m^2^ are used to diagnose DKD in diabetic patients [17].

To ensure accuracy and comparability, all indices were calculated according to the units specified in the original formulas. Among these, TG and HDL‐C were uniformly converted using the following conversion factors: 1 mg/dL = 0.0113 mmol/L (for TG) and 1 mg/dL = 0.0259 mmol/L (for HDL‐C). All conversions were performed before substituting into the formulas to ensure unit consistency across all indices.

The calculation formula of CMI is expressed as follows [18]:

(1) WHtR=WCcmheightcm,CMI=TGmg/dLHDL‐Cmg/dL×WHtR.

The calculation formula of LAP is expressed as follows [19]:

(2) Male: LAP=WCcm−65×TG mmol/L,Female: LAP=WCcm−58×TG mmol/L.

The calculation formula of TyG is expressed as follows [20]:

(3) TyG=LnTG mg/dL×FPGmg/dl2.

The calculation formula of VAI is expressed as follows [15]:

(4) Male: VAI=WC cm39.681.88+×BMI×TG mmol/L1.03×1.31HDL‐C mmol/L,Female: VAI=WC36.581.89+×BMI×TG mmol/L0.81×1.52HDL‐Cmmol/L.

### 2.3. Mortality Assessment

The primary outcome of this study was DKD, and both all‐cause mortality and cardiovascular mortality were also evaluated. The study linked NHANES data with National Death Index (NDI) records to ascertain the mortality status (https://www.cdc.gov/nchs/data-linkage). According to the ICD‐10 coding system, the causes of death for participants included heart disease, cancer, respiratory diseases, accidents, stroke, Alzheimer’s disease, diabetes, influenza, kidney disease, and other various causes. This study defined all‐cause mortality as death due to various factors (including the 10 causes listed above) and cardiovascular mortality as death related to CVDs.

### 2.4. Covariates

This study also considered various potential confounders in the relationship between the four visceral obesity indices and DKD. These factors include demographic variables (age, sex, race, socioeconomic factors, education level [high school or less, some college, college graduate or higher] [21], marital status, and family poverty income ratio [PIR]), BMI, lifestyle factors (alcohol consumption [never, low‐to‐moderate, and heavy], smoking status [never, current, and former]), ALB, low‐density lipoprotein (LDL), TG, HDL, total cholesterol (TC), and uric acid (UA). Medical history variables included hypertension and CVD, where hypertension was defined as self‐reported hypertension, physician‐diagnosed hypertension, or requiring medication treatment. CVD was determined based on self‐reported diagnoses of congestive heart failure, angina, myocardial infarction, or coronary artery disease.

### 2.5. Statistical Analyses

Statistical analyses were performed using Empower Stats (Version 4.1), Stata 17, and R Studio 4.3.0. Given the complex multistage survey design of NHANES, the recommended weighting methods were applied. Weighted logistic regression was used to assess the relationships between CMI, LAP, TyG, VAI, and DKD. The model settings were as follows: Model 1 was unadjusted, Model 2 adjusted for gender, age, and race, and Model 3 further adjusted for education level, marital status, family income, BMI, alcohol consumption, smoking status, hypertension, CVD, ALB, BUN, UA, and TC. In the analysis where VAI is employed as the primary exposure factor, it is essential to emphasize that Model 3 excludes BMI, as the BMI component is already embedded in the calculation formula for VAI. Additionally, a restricted cubic spline (RCS) model was used to analyze whether there is a nonlinear relationship between the four visceral obesity indices and DKD. Subgroup analyses were conducted based on key covariates (e.g., gender, race, family income, hypertension, and CVD) to examine possible interactions between the four visceral obesity indices and different stratification factors. The diagnostic value of each index for DKD was analyzed using receiver operating characteristic (ROC) curves, and mortality was assessed using Kaplan–Meier survival curves. *p* < 0.05 was considered statistically significant.

## 3. Results

### 3.1. Basic Characteristics of Participants

This study included 6722 diabetic patients, of whom 3429 were male and 3293 were female, with a mean age of 49.67 ± 16.03 years. As shown in Table 1, the age of the DKD group was significantly higher than that of the non‐DKD group (*p* < 0.05). In addition, there was a notable difference in gender distribution between the two groups, with a higher proportion of females in the DKD group and a higher proportion of males in the non‐DKD group. The baseline data for gender grouping is shown in Table S1. Other indicators, such as hypertension, CVD, BMI, BUN, and UA, were significantly higher in the DKD group than those in the non‐DKD group, suggesting that metabolic disorders may be associated with DKD. Regarding the four visceral obesity indices, CMI, LAP, TyG, and VAI were significantly higher in the DKD group than those in the non‐DKD group (*p* < 0.05). Specifically, in the DKD group, the proportion of CMI in the Q3 quartile was 45.05%, significantly higher than that of the non‐DKD group; the proportion of LAP in the Q3 quartile was 49.37% in the DKD group, significantly higher than that of the non‐DKD group (30.18%); and the proportions of TyG and VAI in the Q3 quartile in the DKD group were 47.93% and 44.48%, respectively. In conclusion, these indicators are closely associated with the occurrence of DKD.

**TABLE 1:** Basic characteristics of the participants.

	Overall (*n* = 6722)	Non‐DKD (*n* = 5301)	DKD (*n* = 1421)	*p* value
Age (years)	49.67 ± 16.03	46.94 ± 15.06	63.93 ± 13.14	< 0.0001
Gender (%)				0.0233
Male	50.8	51.41	47.64	
Female	49.2	48.59	52.36	
Race (%)				< 0.0001
Mexican American	10.12	10.29	9.27	
Other Hispanic	5.92	6.02	5.4	
Non‐Hispanic White	63.86	64.71	59.41	
Non‐Hispanic Black	12.47	11.36	18.32	
Other/Multiracial	7.62	7.63	7.6	
Education (%)				< 0.0001
Less than 9th grade	8.16	7.43	12.02	
9–11th grade	14.01	13.69	15.68	
High school graduate	23.98	23.4	27.05	
Some college or AA degree	30.1	30.58	27.61	
College graduate or above	23.74	24.91	17.65	
Marital status (%)				< 0.0001
Married/living with partner	63.7	64.64	58.76	
Widowed/divorced/separated	20.41	18.01	32.97	
Never married	15.89	17.35	8.27	
Smoking status (%)				< 0.0001
Never smoker	51.75	52.11	49.83	
Former smoker	26.99	25.15	36.6	
Current smoker	21.26	22.73	13.58	
Drinking status (%)				< 0.0001
Nondrinker	10.35	11.13	6.27	
Low to moderate drinker	18.67	19.44	14.63	
Heavy drinker	28.93	30.93	18.49	
Not clear	42.05	38.5	60.61	
Hypertension (%)				< 0.0001
No	58.78	64.58	28.42	
Yes	41.22	35.42	71.58	
CVD (%)				< 0.0001
NO	94.7	96.33	86.21	
Yes	5.3	3.67	13.79	
PIR (%)	2.87 ± 1.63	2.95 ± 1.63	2.48 ± 1.59	< 0.0001
BMI (kg/m^2^)	29.96 ± 7.15	29.57 ± 6.88	32.01 ± 8.13	< 0.0001
ALB (g/L)	42.47 ± 3.26	42.72 ± 3.18	41.19 ± 3.39	< 0.0001
BUN(mmol/L)	29.96 ± 7.15	29.57 ± 6.88	32.01 ± 8.13	< 0.0001
UA (mg/dL)	5.57 ± 1.44	5.44 ± 1.34	6.25 ± 1.69	< 0.0001
TC (mg/dL)	193.65 ± 43.92	195.20 ± 42.57	185.54 ± 49.62	< 0.0001
Waist (cm)	102.34 ± 17.34	100.93 ± 16.94	109.70 ± 17.56	< 0.0001
WHTR	0.61 ± 0.10	0.60 ± 0.10	0.66 ± 0.10	< 0.0001
eGFR (ml/min/1.73 m^2^)	90.90 ± 22.25	96.22 ± 16.78	63.07 ± 26.28	< 0.0001
CMI	2.81 ± 4.05	2.66 ± 3.94	3.58 ± 4.53	< 0.0001
CMI tertile (%)				< 0.0001
Q1	35.46	37.75	23.47	< 0.0001
Q2	30.82	30.7	31.48	
Q3	33.71	31.55	45.05	
LAP	87.88 ± 103.07	82.39 ± 100.73	116.62 ± 110.20	< 0.0001
LAP tertile (%)				< 0.0001
Q1	35.79	38.75	20.33	
Q2	30.95	31.08	30.3	
Q3	33.26	30.18	49.37	
TyG	9.04 ± 0.84	8.97 ± 0.82	9.41 ± 0.84	< 0.0001
TyG tertile (%)				< 0.0001
Q1	35.83	38.69	20.84	
Q2	32.78	33.08	31.22	
Q3	31.39	28.23	47.93	
VAI	7.32 ± 9.99	6.94 ± 9.64	9.34 ± 11.40	< 0.0001
VAI tertile (%)				< 0.0001
Q1	34.13	36.14	23.64	
Q2	32.27	32.34	31.88	
Q3	33.6	31.52	44.48	

*Note:* ALB: albumin; TyG: triglyceride–glucose index; WHTR, waist‐to‐height ratio.

Abbreviations: BMI, body mass index; BUN, blood urea nitrogen; CMI, cardiometabolic index; CVD, cardiovascular disease; eGFR: estimated glomerular filtration rate; LAP, lipid accumulation product; PIR, poverty income ratio; TC, total cholesterol; UA, uric acid; VAI, visceral adiposity index.

### 3.2. Trend Relationship of Four Visceral Obesity Indices With the Risk of DKD

**FIGURE 2:**
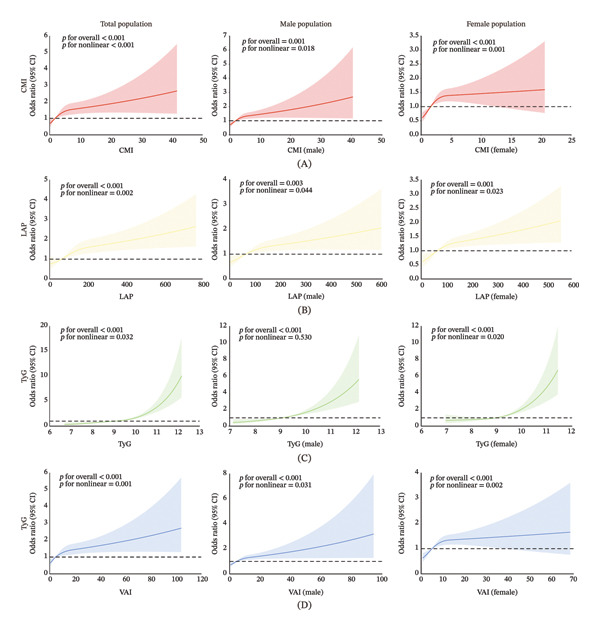
RCS curves between the four visceral obesity indices and DKD. (A) CMI; (B) LAP; (C) TyG; and (D) VAI, adjusted for age, gender, race, education, marital status, smoking status, drinking status, hypertension, CVD, PIR, BMI, ALB, BUN, UA, and TC. The solid line represents the best fit line, and the shaded area represents the 95% confidence interval.

Figure 2 shows the relationship between the four visceral obesity indices and DKD through RCS curves. RCS analysis showed that CMI, LAP, and VAI had a nonlinear relationship with DKD (*P*_nonlinear_ < 0.001), while TyG did not show a nonlinear relationship in the male population (*P*_nonlinear_ = 0.530). This suggests that CMI, LAP, TyG, and VAI have a significant nonlinear association with DKD (Figure 2), whereas the relationship between TyG and DKD in males may be closer to linear. Overall, the association of these indicators with DKD did not show significant sex differences.

### 3.3. Influence of Four Visceral Obesity Indices on DKD Through Multivariate Logistic Regression

#### 3.3.1. Overall

In the total population, TyG was strongly positively correlated with DKD, with a 29.6% increase in DKD risk for every 1‐unit increase (OR = 1.296, *p* = 0.001), and the DKD risk in the Q3 group was significantly higher than that in the Q1 group (OR = 2.319, *p* < 0.001). This suggests that TyG is the most influential indicator among the four visceral obesity indices for DKD. CMI was significantly positively correlated with DKD, with a 4.1% increase in DKD risk for every 1‐unit increase (OR = 1.041, *p* = 0.001), and the DKD risk in the Q3 group was significantly higher than that in the Q1 group (OR = 1.681, *p* < 0.001). VAI was positively correlated with DKD risk, with a 1.8% increase in DKD risk for every 1‐unit increase (OR = 1.018, *p* = 0.004), and the DKD risk in the Q3 group was significantly higher than that in the Q1 group (OR = 1.830, *p* < 0.001). LAP was also positively correlated with DKD risk, with a 0.2% increase in DKD risk for every 1‐unit increase (OR = 1.002, *p* = 0.04), and the disease risk in the Q3 group was significantly higher than that in the Q1 group (OR = 1.792, *p* < 0.001). Although the relative risk increase for LAP was smaller, the risk difference in the higher groups remained significant. The trend test results further support the positive correlation between the four visceral obesity indices and DKD (Table 2).

**TABLE 2:** Regression analysis of CMI, LAP, TyG, and VAI with DKD risk.

Characteristic	Model 1			Model 2			Model 3		
	OR	95% CI	*p* value	OR	95% CI	*p* value	OR	95% CI	*p* value
CMI	1.044	1.022–1.067	< 0.001	1.065	1.039–1.091	< 0.001	1.041	1.018–1.065	0.001
CMI tertiles									
Q1	Ref			Ref			Ref		
Q2	1.650	1.354–2.010	< 0.001	1.313	1.054–1.634	0.015	1.106	0.858–1.426	0.435
Q3	2.297	1.886–2.797	< 0.001	2.301	1.837–2.884	< 0.001	1.681	1.280–2.208	< 0.001
*p* for trend			< 0.001			< 0.001			< 0.001

LAP	1.003	1.002–1.004	< 0.001	1.004	1.002–1.005	< 0.001	1.002	1.000–1.004	0.040
LAP tertiles									
Q1	Ref			Ref			Ref		
Q2	1.50648	1.235–1.837	< 0.001	1.228	0.979–1.541	0.076	1.054	0.803–1.384	0.706
Q3	2.157,277	1.770–2.629	< 0.001	2.583	2.061–3.237	< 0.001	1.792	1.312–2.448	< 0.001
*p* for trend			< 0.001			< 0.001			< 0.001

TyG	1.849	1.688–2.025	< 0.001	1.92	1.710–2.156	< 0.001	1.296	1.105–1.520	0.001
TyG tertiles									
Q1	Ref			Ref			Ref		
Q2	1.752	1.422–2.159	< 0.001	1.369	1.088–1.722	0.007	1.265	0.976–1.067	0.076
Q3	3.151	2.582–3.847	< 0.001	2.733	2.186–3.416	< 0.001	2.319	1.769–3.040	< 0.001
*p* for trend			< 0.001			< 0.001			< 0.001

VAI		1.009–1.029	< 0.001	1.026	1.013–1.038	< 0.001	1.018	1.006–1.031	0.004
VAI tertiles									
Q1	Ref			Ref			Ref		
Q2	1.506	1.235–1.837	< 0.001	1.289	1.035–1.606	0.024	1.181	0.923–1.511	0.187
Q3	2.157	1.770–2.629	< 0.001	2.145	1.713–2.687	< 0.001	1.830	1.403–2.387	< 0.001
*p* for trend			< 0.001			< 0.001			< 0.001

*Note:* Model 1 did not adjust for any confounding factors; Model 2 adjusted for age, gender, and race; Model 3 adjusted for age, gender, race, education, marital status, smoking status, drinking status, hypertension, CVD, PIR, BMI, ALB, BUN, UA, and TC (since the formula for VAI inherently includes BMI, Model 3 does not account for BMI adjustments. However, adjustments for BMI are still applied to other indicators, including CMI, LAP, and TyG).

#### 3.3.2. Stratification by Gender

**FIGURE 3:**
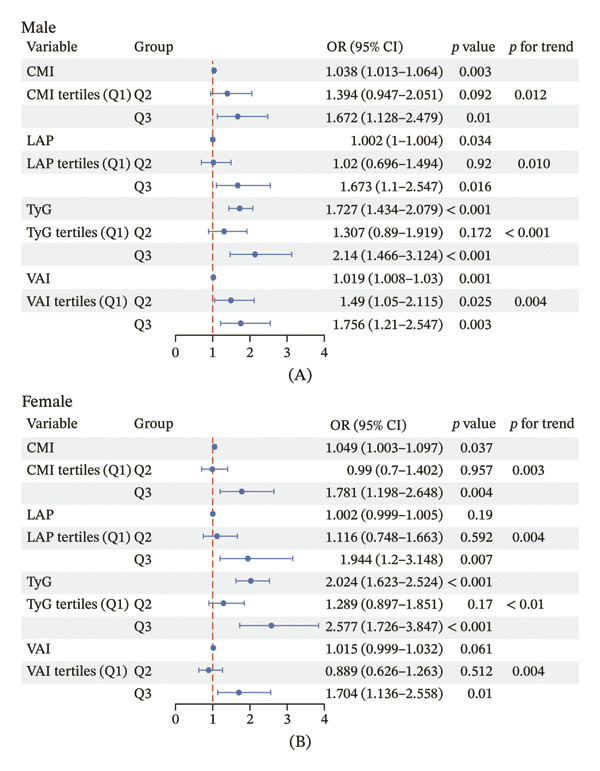
Forest plot of multivariable regression analysis for the four visceral obesity indices and DKD risk (Model 3). (A) Male. (B) Female. Model 3 was adjusted for age, race, education, marital status, smoking status, drinking status, hypertension, CVD, PIR, BMI, ALB, BUN, UA, and TC.

Next, we performed stratified analysis by gender (Tables S2‐S3). Overall, the relationships between CMI, LAP, TyG, VAI, and DKD showed a significant positive correlation in both male and female populations. Longitudinally, for both males and females, TyG had the greatest impact on DKD risk in the Q3 group compared to the Q1 group, followed by LAP, CMI, and VAI in females, and VAI, LAP, and CMI in males. Cross‐sectionally, the risk for females in the CMI Q3 group (OR = 1.781) was higher than that for males in the Q3 group (OR = 1.672), and the risk for females in the LAP Q3 group (OR = 1.944) was higher than that for males in the Q3 group (OR = 1.673). The OR value for TyG in females (OR = 2.024) was higher than that for males (OR = 1.727), and the OR value for TyG in the female Q3 group (2.577) was higher than for males (2.140). This suggests that CMI, LAP, and TyG may have a stronger impact on DKD risk in females. In contrast, the OR value for VAI in the male Q3 group (1.756) was higher than in females (1.704), indicating that VAI may play a more significant role in male DKD risk (Figure 3 **)**.

### 3.4. Subgroup Analysis

To explore the potential association between the four visceral obesity indices and the prevalence of DKD, we performed subgroup analysis, including characteristics such as age, race, PIR, smoking, drinking, CVD, and hypertension (Table S4). The analysis showed that the positive correlation between CMI and DKD was more significant in populations with higher PIR, higher education levels, non‐hypertension, and no CVD (Figure S1 A). For LAP, the OR values in most subgroups were close to 1, indicating that LAP had no significant impact on DKD in these specific subgroups (Figure S1 B). The TyG index was positively correlated with DKD risk in all subgroups, with a more prominent association with increased DKD risk in populations aged under 60, higher education levels, unmarried, current smokers, and those without hypertension and CVD (Figure S1 C). The effect of VAI on DKD was more pronounced in populations aged under 60, with a PIR greater than 3.5, non‐Hispanic Black individuals, higher education levels (especially high school and above), unmarried, never smokers, and those without hypertension (Figure S1 D).

### 3.5. Diagnostic Value of Four Visceral Obesity Indices

**FIGURE 4:**
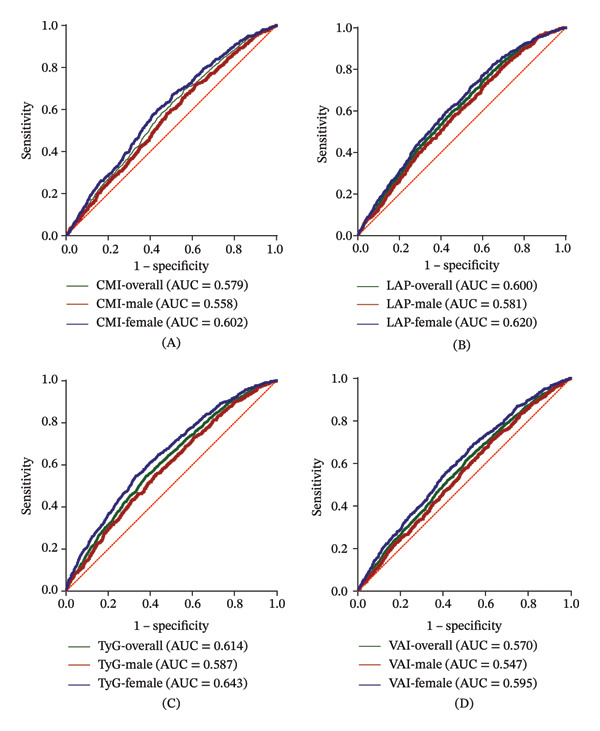
Comparison of ROC curves for predicting DKD using four visceral obesity indices. (A) CMI, (B) LAP, (C) TyG, and (D) VAI.

In the overall population, the area under the curve (AUC) for CMI was 0.579, with AUCs of 0.558 for males and 0.602 for females, suggesting that the risk discriminative ability of CMI is slightly higher in females than in males. The AUC for LAP in the overall population was 0.600, with AUCs of 0.581 for males and 0.620 for females, indicating that the risk identification performance of LAP is superior in females than in males. The AUC for TyG in the overall population was 0.614, with AUCs of 0.587 for males and 0.643 for females, further highlighting a stronger risk association in females. The AUC for VAI in the overall population was 0.570, with AUCs of 0.547 for males and 0.595 for females, also indicating slightly better risk discriminative performance in females than in males (Table 3 and Figure 4).

**TABLE 3:** Comparison of the risk assessment performance of four visceral obesity indices for DKD by gender.

	Group	AUC	95% CI	Cutoff	Sensitivity	Specificity
CMI	Overall	0.579	0.563–0.595	1.461	0.693	0.441
	Male	0.558	0.535–0.581	1.454	0.716	0.387
	Female	0.602	0.579–0.625	1.462	0.671	0.496

LAP	Overall	0.600	0.584–0.616	46.850	0.749	0.395
	Male	0.581	0.558–0.604	45.820	0.733	0.386
	Female	0.620	0.5971–0.642	42.180	0.822	0.353

TyG	Overall	0.614	0.598–0.630	9.217	0.548	0.622
	Male	0.587	0.564–0.610	9.201	0.556	0.573
	Female	0.643	0.620–0.665	9.224	0.548	0.668

VAI	Overall	0.570	0.554–0.587	4.517	0.615	0.494
	Male	0.547	0.524–0.570	3.292	0.709	0.371
	Female	0.595	0.572–0.618	4.312	0.691	0.455

### 3.6. Association of Four Visceral Obesity Indices With All‐Cause and Cardiovascular Mortality

**FIGURE 5:**
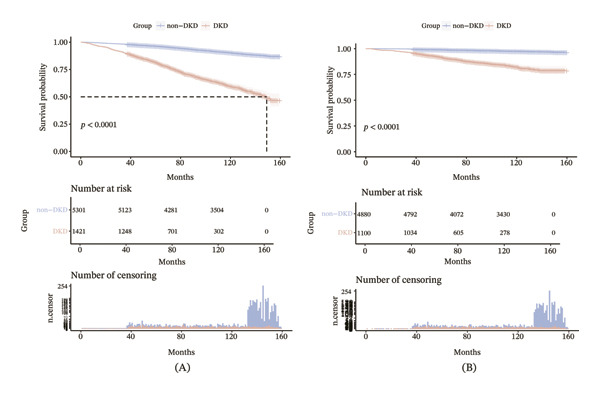
Kaplan–Meier curves for non‐DKD and DKD patients ((A) all‐cause mortality and (B) cardiovascular mortality).

During a median follow‐up period of 133 months, a total of 1009 participants (15.00%) died, of which 267 died from CVD, accounting for 26.46% of the total deaths. The all‐cause mortality (Figure 5A) and cardiovascular mortality (Figure 5B) rates were significantly lower in the non‐DKD group compared to the DKD group.

**FIGURE 6:**
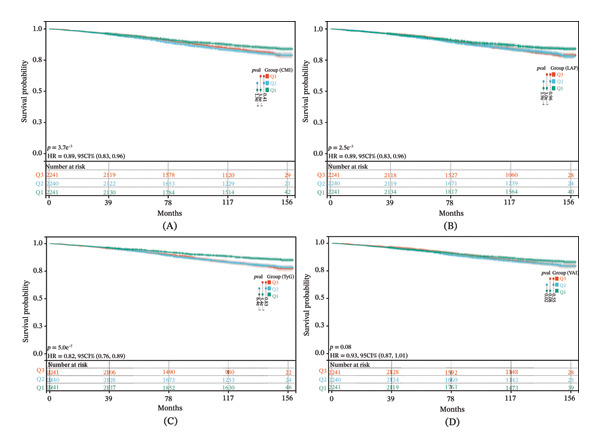
Kaplan–Meier curves for all‐cause mortality of the four visceral obesity indices: ((A) CMI, (B) LAP, (C) TyG, and (D) VAI).

**FIGURE 7:**
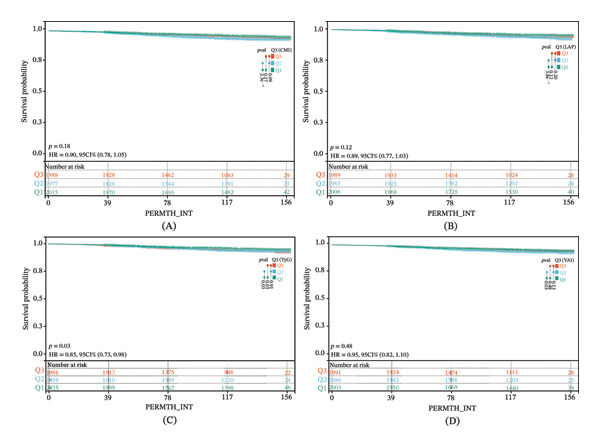
Kaplan–Meier curves for cardiovascular mortality of the four visceral obesity indices ((A) CMI, (B) LAP, (C) TyG, and (D) VAI).

Next, we examined the relationship between the four visceral obesity indices and all‐cause mortality as well as cardiovascular mortality. The results showed that the all‐cause mortality was significantly lower in the low‐level groups (Q1) of CMI, LAP, and TyG compared to the high‐level groups (Q2 and Q3) (*p* < 0.01) (Figure 6A–C). The all‐cause mortality for VAI was significantly lower in the Q1 group compared to the Q2 group (*p* = 0.02) (Figure 6D). Additionally, the cardiovascular mortality for all four indices was significantly lower in the Q1 group compared to the Q2 group (*p* < 0.05) (Figure 7A–D). Further weighted Cox regression analysis revealed that the all‐cause mortality and cardiovascular mortality were higher in the Q2 and Q3 groups compared to the Q1 group. However, this association significantly weakened in Models 2 and 3, and in Model 3, the *p* value did not reach statistical significance (Table 4). This suggests that the relationship between visceral obesity indices and mortality may be influenced by other factors, warranting further investigation.

**TABLE 4:** Cox regression analysis of four visceral obesity indices and mortality.

	Model 1			Model 2			Model 3		
	HR	95% CI	*p* value	HR	95% CI	*p* value	HR	95% CI	*p* value
All‐cause mortality									
CMI tertiles									
Q1	Reference			Reference			Reference		
Q2	1.258	1.152–1.561	0.000	1.055	0–1.152–1.561	0.491	1.010404	0.8627636–1.18331	0.898
Q3	1.341	1.076–1.470	0.004	1.123	0.004–1.076–1.470	0.152	1.009526	0.8503154–1.198548	0.914
LAP tertiles									
Q1	Reference			Reference			Reference		
Q2	1.276	1.097–1.485	0.002	0.906	0.002–1.097–1.485	0.205	0.8990411	0.764542–1.057201	0.198
Q3	1.266	1.084–1.477	0.003	1.020	0.003–1.084–1.477	0.802	0.9279106	0.7708559–1.116964	0.429
TyG tertiles									
Q1	Reference			Reference			Reference		
Q2	1.475	1.264–1.720	0.000	1.144	0–1.264–1.720	0.089	1.14723	0.9788891–1.34452	0.09
Q3	1.492	1.275–1.746	0.000	1.185	0–1.275–1.746	0.036	1.113416	0.9419288–1.316123	0.208
VAI tertiles									
Q1	Reference			Reference			Reference		
Q2	1.202974	1.034–1.399	0.016	1.054	0.016–1.034–1.399	0.495	1.003534	0.8590048–1.172381	0.965
Q3	1.147198	0.984–1.338	0.08	1.150	0.08–0.984–1.338	0.082	1.00512	0.8519936–1.185768	0.952
Cardiovascular mortality									
CMI tertiles									
Q1	Reference			Reference			Reference		
Q2	1.549	1.156–2.077	0.003	1.237	0.921–1.661	0.157	1.118	0.824–1.518	0.474
Q3	1.211	0.886–1.655	0.230	1.140	0.829–1.567	0.419	0.934	0.661–1.318	0.696
LAP tertiles									
Q1	Reference			Reference			Reference		
Q2	1.517	1.132–2.034	0.005	1.085	0.809–1.456	0.586	0.981	0.716–1.344	0.904
Q3	1.254	0.919–1.711	0.153	1.061	0.775–1.453	0.710	0.828	0.571–1.201	0.320
TyG tertiles									
Q1	Reference			Reference			Reference		
Q2	1.341	0.997–1.804	0.052	1.061	0.787–1.435	0.698	1.040	0.765–1.412	0.804
Q3	1.369	1.012–1.852	0.042	1.140	0.839–1.548	0.403	0.994	0.720–1.373	0.973
VAI tertiles									
Q1	Reference			Reference			Reference		
Q2	1.376	1.030–1.839	0.031	1.226	0.915–1.643	0.172	1.127	0.834–1.522	0.436
Q3	1.096	0.805–1.492	0.560	1.154	0.841–1.584	0.375	0.955	0.685–1.331	0.786

*Note:* Model 1 did not adjust for any confounding factors; Model 2 was adjusted for age and race; Model 3 was adjusted for age, race, education, marital status, smoking status, drinking status, hypertension, CVD, PIR, BMI, ALB, BUN, UA, and TC.

## 4. Discussion

**FIGURE 8:**
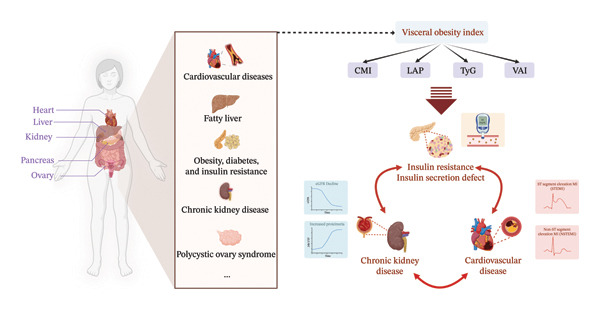
Schematic diagram of the relationship between visceral obesity indices and DKD.

The development of DKD is influenced by the interaction of metabolic abnormalities, kidney damage, and cardiovascular changes, with metabolism serving as the initiating factor. These three factors create a vicious cycle that jointly promotes disease progression [22, 23] (Figure 8). Visceral adipose tissue secretes cytokines and adipokines that induce inflammation, oxidative stress, insulin resistance, and endothelial dysfunction, ultimately impairing kidney structure and function [23]. Dysregulated lipid metabolism accelerates the progression of DKD [24–26]. This study systematically compared the predictive value of four visceral obesity indices (CMI, LAP, TyG, and VAI) in DKD risk prediction using the NHANES database, revealing gender differences and offering new perspectives for the early prevention and control of DKD.

### 4.1. Association Between Visceral Obesity Indices and the Pathophysiology of DKD

In the overall population, we found through RCS analysis that TyG, CMI, VAI, and LAP exhibited a significant nonlinear relationship with DKD (*P*_nonlinear_ < 0.001). This nonlinear relationship may be related to the multifaceted effects of visceral obesity on renal pathophysiology. For example, visceral adipose tissue can secrete various bioactive substances (cytokines, adipokines, etc.) [27], which may increase the risk of DKD by affecting renal hemodynamics and promoting glomerulosclerosis [28]. Adipose tissue can also secrete leptin, which can cause renal dysfunction by promoting sodium excretion and activating sympathetic nervous activity [29]. All four indices were significantly positively correlated with the risk of DKD. Among them, TyG had the greatest impact on DKD, followed by CMI, VAI, and LAP. TyG is a simple index used to assess insulin resistance, calculated from fasting TGs and blood glucose levels [30]. Insulin resistance is an important risk factor for various metabolic diseases, such as T2DM and DKD [31, 32]. Mechanistically, the TyG index is significantly correlated with obesity, glucose metabolism, and inflammation, all of which play crucial roles in the development of DKD. Patients with a history of DKD or atherosclerotic vascular diseases have higher baseline TyG index values, suggesting that the TyG index may increase the risk of DKD by influencing these pathophysiological processes [33]. Furthermore, an elevated TyG index is independently associated with an increased risk of microalbuminuria and a decline in eGFR [34]. A multicenter retrospective cohort study found that compared to patients with lower TyG index values, those with a TyG index reaching or exceeding the critical value had a significantly increased all‐cause mortality in DKD patients, which not only highlights the importance of the TyG index in identifying individuals at higher risk of death among DKD patients but also reflects the close relationship between the TyG index and the severity and prognosis of DKD [35].

CMI is a new indicator for predicting the risk of obesity‐related diseases [36], integrating abdominal obesity and dyslipidemia, the two key metabolic disturbance drivers [18, 37]. It serves as an index for assessing visceral fat distribution and metabolic risk. Visceral adipose tissue secretes cytokines and adipokines that promote insulin resistance, while lipid accumulation in the glomerulus can lead to glomerular injury. CMI is closely related to obesity‐related metabolic disturbances, including diabetes and CVD [13, 38], and increased CMI is associated with a higher risk of DKD [39]. VAI is a gender‐specific index derived from waist circumference, BMI, TG, and HDL, which reflects visceral fat accumulation and dysfunction [15, 40]. VAI has a significant positive correlation with prediabetes and DM, and this association persists after adjusting for confounding variables, showing a nonlinear relationship. These findings support the potential utility of VAI as a predictive tool [41]. Population‐based cohort studies have observed a longitudinal association between VAI and DKD, suggesting that VAI may be independently associated with the incidence of DKD [42]. Cross‐sectional studies indicated that higher VAI is independently associated with increased DKD risk, marking it as a key risk factor for kidney complications in elderly T2DM patients [43]. VAI levels increase with the severity of DKD and serve as an independent predictor of DKD occurrence in T2DM patients [44, 45]. LAP is used to assess excessive central lipid accumulation in adults, reflecting lipid toxicity and the degree of abdominal fat accumulation [19, 46, 47]. When excessive lipids accumulate in the body, they can deposit in the kidneys, triggering oxidative stress, inflammation, and insulin resistance, thereby damaging renal structure and function. Studies have shown that LAP, compared to traditional lipid profiles, has significant predictive ability for CKD and DM [48, 49]. In the field of diabetes, elevated LAP levels are significantly associated with T2DM [50, 51]. In renal research, a study involving 10,012 participants aged ≥ 18 years showed that LAP levels were closely associated with low eGFR and could predict the risk of renal insufficiency [52]. Tang et al. [53] reported higher LAP levels in the DKD group than those in the non‐DKD group and found a positive association between LAP and DKD after adjusting for potential confounders.

Further analysis of the associations between the four visceral obesity indices and mortality in patients with DKD revealed that, after comprehensive adjustment for potential confounding factors such as demographic characteristics, lifestyle factors, and metabolic parameters, the previously observed associations were no longer statistically significant. This attenuation suggests that the influence of obesity indices on mortality risk may be primarily mediated by their associated metabolic abnormalities, including elevated blood pressure, dyslipidemia, hyperuricemia, and obesity itself. In other words, visceral obesity may indirectly affect mortality outcomes by inducing metabolic dysregulation, rather than exerting a direct effect on mortality outcomes [54]. This underscores the necessity of comprehensively evaluating metabolic indicators in clinical practice to optimally manage the prognosis of patients with diabetes.

### 4.2. In‐Depth Interpretation of Gender Differences

In this study, we found that the relationships between CMI, LAP, TyG, and VAI and DKD were significantly positive in both male and female populations, but the risk impacts of different indices varied between genders. Longitudinally, for both males and females, TyG had the greatest impact on DKD risk in the Q3 group compared to the Q1 group, followed by LAP, CMI, and VAI in females and VAI, LAP, and CMI in males. Cross‐sectionally, the risk for females in the Q3 group for CMI, LAP, and TyG was higher than in males, while the risk for VAI in males in the Q3 group was higher than in females. Furthermore, the AUC values for all four indices were higher in females than in males. These gender differences may be related to variations in physiological structure, hormone levels, and metabolic characteristics between males and females.

The distinct calculation formulas of these indices determine their sensitivity to different body composition features. Specifically, VAI incorporates TG and HDL‐C levels, while also adjusting for BMI to refine its assessment. From a physiological perspective, women typically store fat in the hips and thighs, whereas men tend to accumulate fat in the abdominal area [55]. This may allow the VAI to more accurately represent the actual burden of visceral fat in men. In contrast, the CMI, which is based on TGs and HDL‐C, TyG, derived from FPG and TG, and LAP, calculated from waist circumference and TGs, incorporate components that are more closely linked to metabolic syndrome and insulin resistance in women. This relationship likely accounts for their superior predictive performance in the female population.

In addition, female hormone levels, such as estrogen, play an important regulatory role in insulin sensitivity and lipid metabolism. Premenopausal women are protected by estrogen and are less affected by atherosclerotic CVDs and obesity‐related complications. Moreover, gender differences in free fatty acid accumulation and metabolism suggest that females are more capable than males in handling the contribution of visceral fat breakdown to liver nonesterified fatty acid delivery [56]. Related studies also confirm that there is a gender‐specific difference in the critical value of CMI: the critical value for males is much higher than for females, and the correlation between CMI and hyperglycemia and diabetes is stronger in females than in males [18], which is consistent with our findings. For LAP, studies have shown that female participants with CKD, albuminuria, and low eGFR have significantly higher LAP levels than males and that LAP levels in females are significantly positively correlated with CKD prevalence. After adjusting for potential confounders, the CKD prevalence in female participants increased with higher LAP quartiles [57]. On the other hand, in the prediabetes population, after adjusting for confounding factors, the increase in LAP levels was significantly associated with higher urine ACRs in prediabetic females, but not in males, suggesting that females are more sensitive to the effects of LAP on renal function [58]. This gender difference may be attributed to differences in sex hormones, fat distribution, and lifestyle between males and females [57]. Our study also found that the LAP index showed a stronger risk correlation in females, with higher OR values in the Q3 group (1.944 vs. 1.673), and the AUC for LAP in females was higher than in males. The TyG index showed more significant effects in females, with higher OR values in the Q3 group (2.577 vs. 2.140). However, regarding the gender differences in the association between VAI and DKD risk, different studies have reached different conclusions. Wan et al. [22] showed that an increase of 1 standard deviation in VAI was associated with a higher DKD prevalence in females (OR = 1.51; 95% CI: 1.29–1.76, *p* < 0.05), while the effect was not significant in males. However, Li et al. [59] found that a significant VAI–DKD relationship was only evident in males. These differences in findings may be attributed to various factors such as region, sample size, and study design. Our study found that the effect of VAI was slightly stronger in males, but the VAI–AUC was higher in females than in males. This finding calls for the implementation of gender‐specific interventions in clinical practice.

Although the above gender differences were observed in the risk associations, the predictive accuracy of these indices warrants careful consideration. In this study, AUC values for all indices ranged from 0.57 to 0.64, which, despite being statistically significant, indicate limited discriminative ability. These findings suggest that the aforementioned visceral obesity indices are not suitable for standalone diagnosis of DKD. However, given their ease of calculation using routine clinical parameters, they may serve as early screening tools to identify individuals at high risk for DKD. Notably, when combined with sex‐stratified analysis, these indices provide a reference for the development of sex‐specific screening strategies.

### 4.3. Limitations

This study has several limitations: ① It is based on cross‐sectional data from the NHANES database. Although the association between visceral obesity indices (CMI/LAP/TyG/VAI) and DKD was validated through multiple model adjustments, causality cannot be established. Visceral fat accumulation and DKD may have bidirectional effects (e.g., declining kidney function further exacerbating metabolic disturbances), and prospective cohort studies are needed to validate the directional relationship. ② It must be acknowledged that these indicators are surrogate markers derived from anthropometric measurements and biochemical parameters, rather than direct assessments of visceral fat obtained through imaging examinations. Consequently, in specific populations, such as athletes with high muscle mass or elderly individuals with sarcopenia, their accuracy may be compromised, as the relationship between body weight, fat distribution, and metabolic health differs significantly in these groups. ③ Lifestyle factors, such as high‐fat diets and sedentary behavior, are key drivers of visceral fat accumulation but were not sufficiently adjusted for in this study.④ Due to the cross‐sectional nature of the NHANES data, this study was unable to determine whether proteinuria or reduced eGFR represented transient changes or reflected chronic disease, which may limit the interpretation of the results to some extent. We, therefore, recommend that future studies utilize long‐term follow‐up data to more accurately assess the impact of chronic changes in renal function on health outcomes.

### 4.4. Future Research Directions

Future research will include the following: ① Elucidating the mechanisms by conducting prospective nested case–control studies, combining imaging (MRI visceral fat volume) with kidney biomarkers, and dynamically monitoring the temporal relationship between changes in visceral obesity indices and early kidney injury. ② Deepening research on gender heterogeneity: analyzing the regulatory mechanisms of sex hormones (estrogen/testosterone) on visceral fat distribution and adipokine secretion and establishing gender‐specific critical values to guide differentiated interventions. ③ Developing a DKD risk prediction model integrating multiple indices (e.g., CMI + TyG + clinical variables), and validating its efficacy across different ethnic groups and age ranges.

## 5. Conclusion

This study explored the relationship between four visceral obesity indices (CMI, LAP, TyG, and VAI) and DKD risk, with a focus on gender differences. The results show that these indices are significantly positively correlated with DKD risk, with a stronger association and higher risk identification performance observed in females. These findings highlight the importance of considering gender‐specific strategies in DKD screening and prevention.

## Author Contributions

Xuezhe Wang: conceptualization, methodology, writing–original draft, and supervision; Yi Kang: investigation, data curation, and writing–review and editing; Qian Jin: investigation, data curation, and writing–review and editing; Mengqi Zhou: investigation and data curation; Danwen Li: investigation and data curation; Xiaowen Li and Zhenjie Chen: investigation and data curation; Jingwei Zhou, Huijuan Zheng, Jie Lv, and Yaoxian Wang: supervision and funding acquisition.

## Funding

This research was funded by Clinical Research and Achievement Transformation Capability Enhancement Pilot Project of Dongzhimen Hospital, Beijing University of Chinese Medicine (DZMG‐ZLZX‐25022 to Jie Lv), Traditional Chinese Medicine Scientific Research Project of Hebei Provincial Administration of Traditional Chinese Medicine (B2026022 to Jie Lv), Central Universities’ Fundamental Research Funds (2023‐JYB‐JBQN‐020 to Jie Lv), and Joint Project of the China Association of Chinese Medicine (2023DYPLHGG‐11 to Jie Lv).

## Ethics Statement

The NHANES study protocol has been formally approved by the CDC Institutional Review Board, and all participants voluntarily provide written informed consent. Due to the public nature of the NHANES database, research using its data does not require additional Institutional Review Board approval.

## Consent

Please check the Ethics Statement.

## Conflicts of Interest

The authors declare no conflicts of interest.

## Supporting Information

Additional supporting information can be found online in the Supporting Information section.

## Supporting information


**Supporting Information** Supporting 1. Table S1: Baseline characteristics of subjects stratified by gender. Supporting 2. Table S2: Regression analysis of CMI, LAP, TyG, and VAI with DKD risk (male). Supporting 3. Table S3: Regression analysis of CMI, LAP, TyG, and VAI with DKD risk (female). Supporting 4. Table S4: Subgroup analysis. Supporting 5. Figure S1: Forest plot of subgroup analysis (A: CMI; B: LAP; C: TyG; D: VAI).

## Data Availability

Data used to support the findings of this study are available on request from the corresponding authors.
