# Supplementary material for: When Obesity Hits the Kidneys: Sex‐Specific Effects of Visceral Adiposity Indices on Diabetic Kidney Disease Risk
Source: J Nutr Metab. 2026 Jul 20;2026:7057744. doi: 10.1155/jnme/7057744 (PMC13382360; doi:10.1155/jnme/7057744)
Supplement: Supplementary file 1 — Supporting Information Supporting 1. Table S1: Baseline characteristics of subjects stratified by gender. Supporting 2. Table S2: Regression analysis of CMI, LAP, TyG, and VAI with DKD risk (male). Supporting 3. Table S3: Regression analysis of CMI, LAP, TyG, and VAI with DKD risk (female). Supporting 4. Table S4: Subgroup analysis. Supporting 5. Figure S1: Forest plot of subgroup analysis (A: CMI; B: LAP; C: TyG; D: VAI). [file JNME-2026-7057744-s001.docx]

| **1 Table S1 Baseline characteristics of subjects stratified by gender.** | | | | | | |
| --- | --- | --- | --- | --- | --- | --- |
|  | Male (n=3,429) | | | Female (n=3,293) | | |
|  | Non-DKD | DKD | *P* value | Non-DKD | DKD | *P* value |
|  | (n=2,711) | (n=718) |  | (n=2,590) | (n=703) |  |
| Age (years) | 46.52 ± 14.91 | 62.48 ± 13.38 | <0.0001 | 47.39 ± 15.20 | 65.25 ± 12.77 | <0.0001 |
| Race (%) |  |  | 0.0003 |  |  | 0.0001 |
| Mexican American | 10.83 | 10.22 |  | 9.71 | 8.41 |  |
| Other Hispanic | 5.83 | 6.65 |  | 6.23 | 4.26 |  |
| Non-Hispanic White | 65.26 | 57.67 |  | 64.12 | 61 |  |
| Non-Hispanic Black | 9.96 | 16.27 |  | 12.83 | 20.18 |  |
| Other/Multi-Racial | 8.12 | 9.19 |  | 7.1 | 6.16 |  |
| Education (%) |  |  | <0.0001 |  |  | <0.0001 |
| Less than 9th grade | 7.11 | 13.63 |  | 7.77 | 10.55 |  |
| 9-11th grade (Includes 12th grade with no diploma) | 13.84 | 14.79 |  | 13.52 | 16.49 |  |
| High school graduate/GED or equivalent | 23.98 | 25.29 |  | 22.78 | 28.66 |  |
| Some college or AA degree | 28.54 | 24.65 |  | 32.73 | 30.29 |  |
| College graduate or above | 26.52 | 21.63 |  | 23.2 | 14.02 |  |
| Marital_status (%) |  |  | <0.0001 |  |  | <0.0001 |
| Married/Living with partner | 67.54 | 69.98 |  | 61.58 | 48.55 |  |
| Widowed/Divorced/Separated | 13.57 | 21.58 |  | 22.7 | 43.33 |  |
| Never Married | 18.89 | 8.44 |  | 15.72 | 8.12 |  |
| Smoking Status(%) |  |  | <0.0001 |  |  | <0.0001 |
| Never smoker | 44.24 | 43.17 |  | 60.45 | 55.88 |  |
| Former smoker | 29.64 | 39.75 |  | 20.41 | 33.73 |  |
| Current smoker | 26.12 | 17.09 |  | 19.14 | 10.39 |  |
| Drinking state (%) |  |  | <0.0001 |  |  | <0.0001 |
| Non-drinker | 10.19 | 5.26 |  | 12.13 | 7.19 |  |
| low to moderate drinker | 24.71 | 19.92 |  | 13.86 | 9.82 |  |
| heavy drinker | 26.27 | 11.73 |  | 35.86 | 24.63 |  |
| Not clear | 38.83 | 63.08 |  | 38.15 | 58.37 |  |
| Hypertension (%) |  |  | <0.0001 |  |  | <0.0001 |
| NO | 65.06 | 33.36 |  | 64.08 | 23.93 |  |
| Yes | 34.94 | 66.64 |  | 35.92 | 76.07 |  |
| CVD (%) |  |  | <0.0001 |  |  | <0.0001 |
| NO | 94.94 | 84.25 |  | 97.79 | 88 |  |
| Yes | 5.06 | 15.75 |  | 2.21 | 12 |  |
| Poverty Income Ratio (%) | 3.05 ± 1.62 | 2.66 ± 1.63 | <0.0001 | 2.84 ± 1.64 | 2.32 ± 1.55 | <0.0001 |
| BMI (kg/m2) | 29.32 ± 6.19 | 31.80 ± 7.71 | <0.0001 | 29.84 ± 7.52 | 32.19 ± 8.50 | <0.0001 |
| ALB (g/L) | 43.70 ± 3.07 | 41.60 ± 3.37 | <0.0001 | 41.67 ± 2.95 | 40.82 ± 3.37 | <0.0001 |
| BUN(mmol/L) | 4.79 ± 1.48 | 7.12 ± 3.68 | <0.0001 | 4.24 ± 1.44 | 7.05 ± 3.45 | <0.0001 |
| UA (mg/dL) | 5.98 ± 1.25 | 6.46 ± 1.70 | <0.0001 | 4.86 ± 1.19 | 6.05 ± 1.66 | <0.0001 |
| TC (mg/dL) | 192.30 ± 42.87 | 177.43 ± 51.26 | <0.0001 | 198.27 ± 42.04 | 192.92 ± 46.87 | 0.0072 |
| WAIST (cm) | 103.40 ± 16.29 | 112.56 ± 17.19 | <0.0001 | 98.32 ± 17.22 | 107.10 ± 17.49 | <0.0001 |
| WHTR | 0.59 ± 0.09 | 0.65 ± 0.09 | <0.0001 | 0.61 ± 0.11 | 0.67 ± 0.11 | <0.0001 |
| eGFR | 96.38 ± 16.56 | 66.66 ± 26.19 | <0.0001 | 96.05 ± 17.00 | 59.80 ± 25.94 | <0.0001 |
| CMI | 3.18 ± 4.46 | 4.01 ± 5.24 | 0.0001 | 2.12 ± 3.20 | 3.19 ± 3.72 | <0.0001 |
| CMI tertile (%) |  |  | <0.0001 |  |  | <0.0001 |
| Q1 | 31.21 | 20.56 |  | 44.68 | 26.12 |  |
| Q2 | 30.86 | 31.69 |  | 30.53 | 31.29 |  |
| Q3 | 37.94 | 47.75 |  | 24.79 | 42.58 |  |
| LAP | 89.58 ± 100.62 | 117.87 ± 115.96 | <0.0001 | 74.78 ± 100.28 | 115.49 ± 104.67 | <0.0001 |
| LAP tertile (%) |  |  | <0.0001 |  |  | <0.0001 |
| Q1 | 36.12 | 22.3 |  | 41.52 | 18.54 |  |
| Q2 | 30.95 | 29.07 |  | 31.21 | 31.43 |  |
| Q3 | 32.93 | 48.64 |  | 27.27 | 50.03 |  |
| TyG | 9.11 ± 0.82 | 9.45 ± 0.88 | <0.0001 | 8.83 ± 0.78 | 9.37 ± 0.80 | <0.0001 |
| TyG tertile (%) |  |  | <0.0001 |  |  | <0.0001 |
| Q1 | 32.47 | 20.35 |  | 45.26 | 21.29 |  |
| Q2 | 33.61 | 30.61 |  | 32.51 | 31.78 |  |
| Q3 | 33.92 | 49.04 |  | 22.22 | 46.93 |  |
| VAI | 7.21 ± 9.53 | 8.80 ± 11.44 | 0.0008 | 6.65 ± 9.75 | 9.84 ± 11.34 | <0.0001 |
| VAI tertile (%) |  |  | <0.0001 |  |  | <0.0001 |
| Q1 | 36.37 | 25.99 |  | 35.89 | 21.5 |  |
| Q2 | 33.74 | 29.71 |  | 32.34 | 31.88 |  |
| Q3 | 30.36 | 48.79 |  | 31.52 | 44.48 |  |

CVD: cardiovascular disease; PIR:poverty income ratio; BMI: body mass index; ALB: albumin; BUN:blood urea nitrogen; UA :uric acid; TC:total cholesterol; WHTR: waist-to-height ratio; eGFR: estimated glomerular filtration rate; CMI:cardiometabolic index; LAP: lipid accumulation product; LAP: lipid accumulation product; TyG:triglyceride-glucose index; VAI: visceral adiposity index.

**2 Stratification by Gender**

Next, we performed stratified analysis by gender. The results showed that all four indices were positively correlated with DKD risk in males. For CMI, every 1-unit increase was associated with a 3.8% increase in DKD risk (OR=1.038, *P*=0.003), and the DKD risk in the Q3 group was significantly higher than in the Q1 group (OR=1.672, *P*=0.010). For LAP, every 1-unit increase was associated with a 0.2% increase in DKD risk (OR=1.002, *P*=0.034), and the DKD risk in the Q3 group was significantly higher than in the Q1 group (OR=1.673, *P*=0.016). For TyG, every 1-unit increase was associated with a 72.7% increase in DKD risk (OR=1.727, P<0.001). Analysis by TyG quartiles showed that the DKD risk in the Q3 group was significantly higher than in the Q1 group (OR=2.14, P<0.001). For VAI, every 1-unit increase was associated with a 1.9% increase in DKD risk (OR=1.019, P=0.001) (**Table S2**).

In females, all four indices were positively correlated with DKD risk. For CMI, every 1-unit increase was associated with a 4.9% increase in DKD risk (OR=1.049, *P*=0.037), and the DKD risk in the Q3 group was significantly higher than in the Q1 group (OR=1.781, *P*=0.004). For LAP, every 1-unit increase was associated with a 0.2% increase in DKD risk (OR=1.002, *P*=0.19), and the DKD risk in the Q3 group was significantly higher than in the Q1 group (OR=1.944, *P*=0.007). The relationship between TyG and DKD was significant in females (OR=2.024, *P*<0.001), with the DKD risk in the Q3 group being significantly higher than in the Q1 group (OR=2.577, P<0.001). For VAI, every 1-unit increase was associated with a 1.5% increase in DKD risk (OR=1.015, P=0.061), and the DKD risk in the Q3 group was significantly higher than in the Q1 group (OR=1.704, *P*=0.01) (Table S3).

**Table S2 Regression analysis of CMI, LAP, TyG, and VAI with DKD Risk (Male)**

| Male | Model 1 | | | Model 2 | | | Model 3 | | |
| --- | --- | --- | --- | --- | --- | --- | --- | --- | --- |
|  | OR | 95% CI | p-value | OR | 95% CI | p-value | OR | 95% CI | p-value |
| CMI | 1.031 | 1.012-1.051 | 0.002 | 1.053 | 1.031-1.074 | <0.01 | 1.038 | 1.013-1.064 | 0.003 |
| CMI tertiles |  |  |  |  |  |  |  |  |  |
| Q1 | Ref | | | Ref | | | Ref | | |
| Q2 | 1.559 | 1.156-2.103 | 0.004 | 1.332 | 0.957-1.854 | 0.089 | 1.394 | 0.947-2.051 | 0.092 |
| Q3 | 1.911 | 1.428-2.557 | <0.001 | 2.093 | 1.504-2.911 | <0.001 | 1.672 | 1.128-2.479 | 0.010 |
| *P* for trend |  |  | <0.001 |  |  | <0.001 |  |  | 0.012 |
| LAP | 1.002 | 1.001-1.003 | <0.001 | 1.003 | 1.002-1.005 | <0.001 | 1.002 | 1.000-1.004 | 0.034 |
| LAP tertiles |  |  |  |  |  |  |  |  |  |
| Q1 | Ref | | | Ref | | | Ref | | |
| Q2 | 1.521 | 1.136-2.038 | 0.005 | 1.105 | 0.801-1.524 | 0.543 | 1.02 | 0.696-1.494 | 0.920 |
| Q3 | 2.393 | 1.798-3.184 | <0.001 | 2.312 | 1.687-3.169 | <0.01 | 1.673 | 1.100-2.547 | 0.016 |
| *P* for trend |  |  | <0.001 |  |  |  |  |  | 0.01 |
| TyG | 1.595 | 1.403-1.814 | <0.001 | 1.732 | 1.481-2.026 | <0.01 | 1.727 | 1.434-2.079 | <0.001 |
| TyG tertiles |  |  |  |  |  |  |  |  |  |
| Q1 | Ref | | | Ref | | | Ref | | |
| Q2 | 1.453 | 1.068-1.976 | 0.017 | 1.321 | 0.944-1.85 | 0.105 | 1.307 | 0.89-1.919 | 0.172 |
| Q3 | 2.307 | 1.727-3.081 | <0.001 | 2.338 | 1.696-3.223 | <0.001 | 2.140 | 1.466-3.124 | <0.001 |
| *P* for trend |  |  | <0.001 |  |  | <0.001 |  |  | <0.001 |
| VAI | 1.013 | 1.004-1.022 | 0.003 | 1.023 | 1.014-1.032 | <0.001 | 1.019 | 1.008-1.03 | 0.001 |
| VAI tertiles |  |  |  |  |  |  |  |  |  |
| Q1 | Ref | | | Ref | | | Ref | | |
| Q2 | 1.545 | 1.17-2.04 | 0.002 | 1.464 | 1.075-1.994 | 0.016 | 1.490 | 1.05-2.115 | 0.025 |
| Q3 | 1.706 | 1.281-2.271 | <0.001 | 2.005 | 1.452-2.768 | <0.001 | 1.756 | 1.21-2.547 | 0.003 |
| *P* for trend |  |  | <0.001 |  |  | <0.001 |  |  | 0.004 |

Model 1 did not adjust for any confounding factors; Model 2 adjusted for age, race; Model 3 adjusted for age, race, education, marital status, smoking status, drinking status, hypertension, CVD, PIR, BMI, ALB, BUN, UA and TC.

**Table S3 Regression analysis of CMI, LAP, TyG, and VAI with DKD Risk (Female)**

| Female | Model 1 |  |  | Model 2 |  |  | Model 3 |  |  |
| --- | --- | --- | --- | --- | --- | --- | --- | --- | --- |
|  | OR | 95% CI | p-value | OR | 95% CI | p-value | OR | 95% CI | p-value |
| CMI | 1.091 | 1.022-1.165 | 0.009 | 1.098 | 1.014-1.188 | 0.021 | 1.049 | 1.003-1.097 | 0.037 |
| CMI tertiles |  |  |  |  |  |  |  |  |  |
| Q1 | Ref | | | Ref | | | Ref | | |
| Q2 | 1.753 | 1.347-2.282 | <0.001 | 1.301 | 0.97-1.746 | 0.079 | 0.990 | 0.700-1.402 | 0.957 |
| Q3 | 2.938 | 2.24-3.854 | <0.001 | 2.54 | 1.856-3.477 | <0.001 | 1.781 | 1.198-2.648 | 0.004 |
| *P* for trend |  |  | <0.001 |  |  | <0.001 |  |  | 0.003 |
| LAP | 1.004 | 1.002-1.007 | <0.001 | 1.004 | 1.001-1.007 | 0.004 | 1.002 | 0.999-1.005 | 0.19 |
| LAP tertiles |  |  |  |  |  |  |  |  |  |
| Q1 | Ref | | | Ref | | | Ref | | |
| Q2 | 2.255 | 1.685-3.019 | <0.001 | 1.373 | 0.995-1.895 | 0.054 | 1.116 | 0.748-1.663 | 0.592 |
| Q3 | 4.109 | 3.083-5.477 | <0.001 | 2.913 | 2.098-4.044 | <0.001 | 1.944 | 1.2-3.148 | 0.007 |
| *P* for trend |  |  | <0.001 |  |  | <0.001 |  |  | 0.004 |
| TyG | 2.311 | 2.008-2.66 | <0.001 | 2.172 | 1.825-2.585 | <0.001 | 2.024 | 1.623-2.524 | <0.001 |
| TyG tertiles |  |  |  |  |  |  |  | - |  |
| Q1 | Ref | | | Ref | | | Ref | | |
| Q2 | 2.079 | 1.565-2.762 | <0.001 | 1.413 | 1.031-1.938 | 0.032 | 1.289 | 0.897-1.851 | 0.17 |
| Q3 | 4.491 | 3.404-5.924 | <0.001 | 3.189 | 2.327-4.372 | <0.001 | 2.577 | 1.726-3.847 | <0.001 |
| *P* for trend |  |  | <0.001 |  |  | <0.001 |  |  | <0.01 |
| VAI | 1.028 | 1.005-1.051 | 0.018 | 1.03 | 1.001-1.059 | 0.04 | 1.015 | 0.999-1.032 | 0.061 |
| VAI tertiles |  |  |  |  |  |  |  |  |  |
| Q1 | Ref | | | Ref | | | Ref | | |
| Q2 | 1.47 | 1.107-1.952 | 0.008 | 1.143 | 0.836-1.563 | 0.401 | 0.889 | 0.626-1.263 | 0.512 |
| Q3 | 2.682 | 2.034-3.537 | <0.001 | 2.27 | 1.645-3.132 | <0.001 | 1.704 | 1.136-2.558 | 0.01- |
| *P* for trend | 1.657 | 1.441-1.906 | <0.001 |  |  | <0.001 |  |  | 0.004 |

Model 1 did not adjust for any confounding factors; Model 2 adjusted for age, race; Model 3 adjusted for age, race, education, marital status, smoking status, drinking status, hypertension, CVD, PIR, BMI, ALB, BUN, UA and TC.

**Table S4 Subgroup analysis**

|  | CMI | | | LAP | | | TyG | | | VAI | | |
| --- | --- | --- | --- | --- | --- | --- | --- | --- | --- | --- | --- | --- |
| Subgroup | P value | P for interaction | OR（95%CI） | P value | P for interaction | OR（95%CI） | P value | P for interaction | OR（95%CI） | P value | P for interaction | OR（95%CI） |
| Age |  | 0.456 |  |  | 0.047 |  | <0.001 |  | 2.33 (2.07 ~ 2.62) | 2.33 | 0.31 |  |
| <60 years | <0.001 |  | 1.04 (1.03 ~ 1.06) | <.001 |  | 1.00 (1.00 ~ 1.00) | <0.001 |  | 1.22 (1.10 ~ 1.36) | 1.22 |  | 1.02 (1.01 ~ 1.03) |
| ≥60 years | 0.019 |  | 1.03 (1.01 ~ 1.06) | 0.002 |  | 1.00 (1.00 ~ 1.00) |  | 0.802 |  | 1 |  | 1.01 (1.00 ~ 1.02) |
| PIR |  | 0.219 |  |  | 0.267 |  | <0.001 |  | 1.56 (1.39 ~ 1.75) | 1.56 | 0.202 |  |
| 0-1.3 | 0.029 |  | 1.02 (1.00 ~ 1.04) | <.001 |  | 1.00 (1.00 ~ 1.00) | <0.001 |  | 1.56 (1.40 ~ 1.75) | 1.56 |  | 1.01 (1.00 ~ 1.02) |
| 1.3-3.5 | 0.071 |  | 1.02 (1.00 ~ 1.04) | 0.003 |  | 1.00 (1.00 ~ 1.00) | <0.001 |  | 1.66 (1.41 ~ 1.95) | 1.66 |  | 1.01 (1.00 ~ 1.02) |
| >3.5 | 0.002 |  | 1.05 (1.02 ~ 1.09) | <.001 |  | 1.00 (1.00 ~ 1.00) |  | 0.516 |  | 1 |  | 1.02 (1.01 ~ 1.04) |
| Race |  | 0.002 |  |  | 0.004 |  | <0.001 |  | 1.71 (1.44 ~ 2.03) | 1.71 | <0.001 |  |
| Mexican American | 0.32 |  | 1.01 (0.99 ~ 1.04) | 0.007 |  | 1.00 (1.00 ~ 1.00) | <0.001 |  | 1.78 (1.42 ~ 2.23) | 1.78 |  | 1.01 (1.00 ~ 1.02) |
| Other Hispanic | 0.088 |  | 1.03 (1.00 ~ 1.06) | 0.191 |  | 1.00 (1.00 ~ 1.00) | <0.001 |  | 1.83 (1.62 ~ 2.07) | 1.83 |  | 1.01 (1.00 ~ 1.02) |
| Non-Hispanic White | <0.001 |  | 1.04 (1.02 ~ 1.07) | <.001 |  | 1.00 (1.00 ~ 1.00) | <0.001 |  | 1.74 (1.50 ~ 2.01) | 1.74 |  | 1.02 (1.01 ~ 1.03) |
| Non-Hispanic Black | <0.001 |  | 1.15 (1.08 ~ 1.21) | <.001 |  | 1.01 (1.00 ~ 1.01) | 0.005 |  | 1.42 (1.11 ~ 1.82) | 1.42 |  | 1.06 (1.03 ~ 1.08) |
| Other/Multi-Racial | 0.554 |  | 1.01 (0.97 ~ 1.06) | 0.164 |  | 1.00 (1.00 ~ 1.00) |  | 0.002 |  | 1 |  | 1.00 (0.99 ~ 1.02) |
| Education |  | 0.115 |  |  | <.001 |  | 0.05 |  | 1.19 (1.00 ~ 1.42) | 1.19 | 0.032 |  |
| Less than 9th grade | 0.693 |  | 0.99 (0.96 ~ 1.03) | 0.972 |  | 1.00 (1.00 ~ 1.00) | <0.001 |  | 1.55 (1.31 ~ 1.83) | 1.55 |  | 1.00 (0.98 ~ 1.01) |
| 9-11th grade | 0.061 |  | 1.03 (1.00 ~ 1.06) | <.001 |  | 1.00 (1.00 ~ 1.00) | <0.001 |  | 1.55 (1.34 ~ 1.80) | 1.55 |  | 1.01 (1.00 ~ 1.02) |
| High school graduate | 0.147 |  | 1.02 (0.99 ~ 1.06) | <.001 |  | 1.00 (1.00 ~ 1.00) | <0.001 |  | 1.87 (1.62 ~ 2.15) | 1.87 |  | 1.01 (1.00 ~ 1.03) |
| Some college or AA degree | 0.001 |  | 1.04 (1.01 ~ 1.06) | <.001 |  | 1.00 (1.00 ~ 1.00) | <0.001 |  | 1.74 (1.44 ~ 2.11) | 1.74 |  | 1.02 (1.01 ~ 1.03) |
| College graduate or above | 0.01 |  | 1.06 (1.01 ~ 1.11) | <.001 |  | 1.00 (1.00 ~ 1.01) |  | <.001 |  | 1 |  | 1.02 (1.00 ~ 1.04) |
| Marital status |  | <0.001 |  |  | <.001 |  | <0.001 |  | 1.61 (1.46 ~ 1.77) | 1.61 | <0.001 |  |
| Married/Living with partner | 0.002 |  | 1.03 (1.01 ~ 1.04) | <.001 |  | 1.00 (1.00 ~ 1.00) | <0.001 |  | 1.34 (1.18 ~ 1.53) | 1.34 |  | 1.01 (1.00 ~ 1.02) |
| Widowed/Divorced/Separated | 0.511 |  | 1.01 (0.98 ~ 1.03) | 0.416 |  | 1.00 (1.00 ~ 1.00) | <0.001 |  | 2.42 (1.94 ~ 3.02) | 2.42 |  | 1.01 (0.99 ~ 1.02) |
| Never Married | <0.001 |  | 1.15 (1.09 ~ 1.21) | <.001 |  | 1.01 (1.01 ~ 1.01) |  | 0.013 |  | 1 |  | 1.05 (1.03 ~ 1.08) |
| Smoking status |  | 0.208 |  |  | 0.262 |  | <0.001 |  | 1.76 (1.59 ~ 1.95) | 1.76 | 0.473 |  |
| Never smoker | <0.001 |  | 1.04 (1.02 ~ 1.07) | <.001 |  | 1.00 (1.00 ~ 1.00) | <0.001 |  | 1.38 (1.21 ~ 1.57) | 1.38 |  | 1.02 (1.01 ~ 1.03) |
| Former smoker | 0.333 |  | 1.01 (0.99 ~ 1.04) | 0.003 |  | 1.00 (1.00 ~ 1.00) | <0.001 |  | 1.58 (1.33 ~ 1.87) | 1.58 |  | 1.01 (1.00 ~ 1.02) |
| Current smoker | 0.027 |  | 1.03 (1.00 ~ 1.05) | 0.007 |  | 1.00 (1.00 ~ 1.00) |  | 0.742 |  | 1 |  | 1.01 (1.00 ~ 1.02) |
| Drinking status |  | 0.592 |  |  | 0.968 |  | <0.001 |  | 1.64 (1.26 ~ 2.14) | 1.64 | 0.716 |  |
| Non-drinker | 0.776 |  | 1.01 (0.95 ~ 1.08) | 0.087 |  | 1.00 (1.00 ~ 1.01) | <0.001 |  | 1.47 (1.22 ~ 1.75) | 1.47 |  | 1.01 (0.98 ~ 1.03) |
| low to moderate drinker | 0.48 |  | 1.01 (0.98 ~ 1.05) | 0.01 |  | 1.00 (1.00 ~ 1.00) | <0.001 |  | 1.46 (1.26 ~ 1.70) | 1.46 |  | 1.00 (0.99 ~ 1.02) |
| heavy drinker | 0.376 |  | 1.01 (0.98 ~ 1.05) | 0.007 |  | 1.00 (1.00 ~ 1.00) | <0.001 |  | 1.58 (1.43 ~ 1.75) | 1.58 |  | 1.01 (1.00 ~ 1.02) |
| Not clear | <0.001 |  | 1.03 (1.01 ~ 1.05) | <.001 |  | 1.00 (1.00 ~ 1.00) |  | <.001 |  | 1 |  | 1.01 (1.01 ~ 1.02) |
| Hypertension |  | 0.192 |  |  | 0.776 |  | <0.001 |  | 1.86 (1.65 ~ 2.11) | 1.86 | 0.221 |  |
| NO | 0.001 |  | 1.03 (1.01 ~ 1.05) | 0.004 |  | 1.00 (1.00 ~ 1.00) | <0.001 |  | 1.26 (1.15 ~ 1.38) | 1.26 |  | 1.01 (1.00 ~ 1.02) |
| Yes | 0.254 |  | 1.01 (0.99 ~ 1.03) | 0.009 |  | 1.00 (1.00 ~ 1.00) |  | <.001 |  | 1 |  | 1.01 (1.00 ~ 1.01) |
| CVD |  | 0.167 |  |  | 0.056 |  | <0.001 |  | 1.65 (1.53 ~ 1.78) | 1.65 | 0.588 |  |
| NO | <0.001 |  | 1.03 (1.01 ~ 1.04) | <.001 |  | 1.00 (1.00 ~ 1.00) | 0.962 |  | 1.01 (0.79 ~ 1.28) | 1.01 |  | 1.01 (1.01 ~ 1.02) |
| Yes | 0.665 |  | 0.99 (0.93 ~ 1.04) | 0.781 |  | 1.00 (1.00 ~ 1.00) |  |  |  | 0.584 |  | 1.01 (0.98 ~ 1.03) |

**
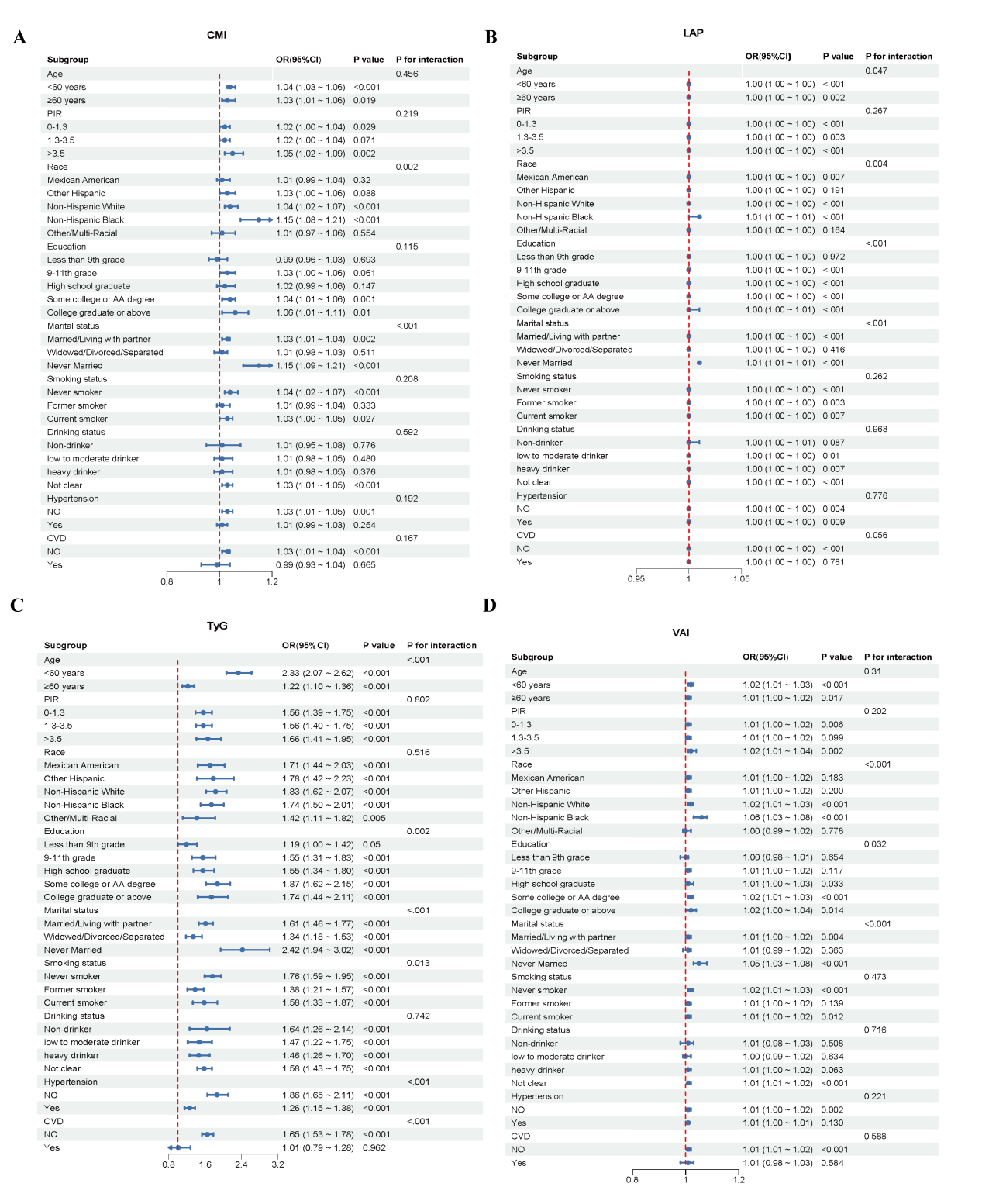
**

**Figure S1 Forest plot of subgroup analysis (A: CMI; B: LAP; C: TyG; D: VAI)**
